# Reply to the comment by Sakamoto et al. on “The method to identify invasive mechanical ventilation with Japanese claim data”

**DOI:** 10.1186/s40560-024-00767-7

**Published:** 2024-12-23

**Authors:** Hiroyuki Ohbe, Nobuaki Shime, Hayato Yamana, Tadahiro Goto, Yusuke Sasabuchi, Daisuke Kudo, Hiroki Matsui, Hideo Yasunaga, Shigeki Kushimoto

**Affiliations:** 1 Department of Emergency and Critical Care Medicine, Tohoku University Hospital, 1-1 Seiryo-Machi, Aoba-Ku, Sendai, 980-8574 Japan; 2 Department of Clinical Epidemiology and Health Economics, School of Public Health, The University of Tokyo, 7-3-1 Hongo, Bunkyo-Ku, Tokyo, 113-0033 Japan; 3 Department of Emergency and Critical Care Medicine, Graduate School of Biomedical and Health Sciences, Hiroshima University, 1-2-3 Kasumi, Minami-Ku, Hiroshima, 734-8551 Japan; 4 Data Science Center, Jichi Medical University, 3311-1 Yakushiji, Shimotsuke, Tochigi 329-0498 Japan; 5 TXP Medical Co., Ltd., 41-1 H1O Kanda 706, Kanda Higashimatsushita-Cho, Chiyoda-Ku, Tokyo, 101-0042 Japan; 6 Department of Real-World Evidence, Graduate School of Medicine, The University of Tokyo, 7-3-1 Hongo, Bunkyo-Ku, Tokyo, 113-0033 Japan; 7 Division of Emergency and Critical Care Medicine, Tohoku University Graduate School of Medicine, 2-1 Seiryo-Machi, Aoba-Ku, Sendai, Miyagi 980-8575 Japan; 8 Department of Health Services Research, Graduate School of Medicine, The University of Tokyo, 7-3-1 Hongo, Bunkyo-Ku, Tokyo, 113-0033 Japan

To the Editor,

We sincerely appreciate Sakamoto et al. for their thoughtful feedback [1] and for raising important concerns regarding our recently published work in *Journal of Intensive Care* [2]. In their Letter to the Editor [1], the authors expressed concerns regarding the method to identify patients requiring invasive mechanical ventilation using Japanese administrative claims data, including health insurance claims data and Diagnosis Procedure Combination inpatient (DPC) data.

We acknowledge that studies using administrative claims databases rely on data not originally intended for research purposes, making validation studies critical to quantify the reliability and accuracy of such definitions. A previous validation study of Japanese DPC data showed that the sensitivity and specificity of major procedures exceeded 90% [3]. Since procedural claims are linked to hospital reimbursements, hospitals have a strong incentive to file them accurately, which may explain the high sensitivity and specificity observed in that study. However, no previous studies have validated the identification of patients requiring invasive mechanical ventilation based on Japanese administrative claims data.

The Japanese procedure code “J045 Artificial ventilation” included 20 reimbursement codes for various forms of artificial ventilation (Table 1). Of these, the codes 140,039,550 and 140,039,650 correspond to noninvasive mechanical ventilation, labeled as “Mechanical ventilation (nasal mask ventilator).” In all DPC database studies conducted by Ohbe et al., the definition of invasive mechanical ventilation excluded codes associated with noninvasive mechanical ventilation (please refer to Table 1 for the definition of invasive mechanical ventilation) [4, 5].

**Table 1:** Lists of reimbursement codes for the Japanese procedure code “J045 Artificial ventilation” from April 1, 2018, through April 1, 2019

Kubun code	Reimbursement code	Reimbursement name in English	Medical	Definition of IMV
			fee points	
J045	140,009,310^a^	Mechanical ventilation	302	Yes
J045	140,023,510	Mechanical ventilation (beyond 5 h up to day 14)	950	Yes
J045	140,039,550^a^	Mechanical ventilation (nasal mask ventilator)	302	No
J045	140,039,650	Mechanical ventilation (nasal mask ventilator, beyond 5 h up to day 14)	950	No
J045	140,009,550^a^	Mechanical ventilation (closed-circuit anesthesia machine)	302	Yes
J045	140,023,750	Mechanical ventilation (closed-circuit anesthesia machine, beyond 5 h up to day 14)	950	Yes
J045	140,009,750^a^	Mechanical ventilation (semi-closed circuit anesthesia machine)	302	Yes
J045	140,023,950	Mechanical ventilation (semi-closed circuit anesthesia machine, beyond 5 h up to day 14)	950	Yes
J045	140,009,650^a^	Oxygen inhalation (micro adapter)	302	Yes
J045	140,023,850	Oxygen inhalation (micro adapter, beyond 5 h up to day 14)	950	Yes
J045	140,039,850*	Oxygen inhalation with endotracheal intubation using closed-circuit anesthesia machine	302	Yes
J045	140,039,950	Oxygen inhalation with endotracheal intubation using closed-circuit anesthesia Machine (beyond 5 h up to day 14)	950	Yes
J045	140,009,450^a^	Anhydrous alcohol inhalation therapy	302	Yes
J045	140,023,650	Anhydrous alcohol inhalation therapy (beyond 5 h up to day 14)	950	Yes
J045	140,009,950^a^	Oxygen pressurization (endotracheal intubation with closed-circuit anesthesia machine)	302	Yes
J045	140,024,150	Oxygen pressurization (endotracheal intubation with closed-circuit anesthesia machine, beyond 5 h up to day 14)	950	Yes
J045	140,010,050^b^	Continuous positive airway pressure (CPAP)	302	No
J045	140,024,250^b^	Continuous positive airway pressure (CPAP, beyond 5 h up to day 14)	950	No
J045	140,010,150^b^	Intermittent mandatory ventilation	302	Yes
J045	140,024,350^b^	Intermittent mandatory ventilation (beyond 5 h up to day 14)	950	Yes

IMV, invasive mechanical ventilation

^a^These codes were reimbursed for up to 30 min. If the duration was more than 30 min and up to 5 h, these codes were reimbursed at 302 points, with an additional 50 points for each subsequent 30-min period

^b^These codes are only applicable to neonates

Sakamoto et al. correctly pointed out that “artificial ventilation during cardiopulmonary resuscitation (CPR) can be claimed by J045”, highlighting an error in our methods section, where we stated that “invasive mechanical ventilation during CPR were excluded.” Patients who received artificial ventilation solely during CPR—specifically those who either died without achieving return of spontaneous circulation (ROSC) or did not require artificial ventilation after ROSC—could have caused bias in our study. To address this concern, we conducted a post-hoc analysis, excluding 16,510 patients who received CPR on the day of artificial ventilation initiation. The main results were consistent with those of the primary analysis (Table 2).

**Table 2:** Results of the sensitivity analyses excluding 16,510 patients who received cardiopulmonary resuscitation on the day of invasive mechanical ventilation initiation

Statistic	Model 1	Model 2	Model 3
Hospital level			
ICC (%)	82.5 (79.2–85.3)	82.2 (78.9–85.1)	16.9 (13.9–20.3)
MOR	42.8 (25.9–59.8)	41.2 (25.2–57.2)	2.18 (1.99–2.38)
PCV (%)			
Models 1 and 2	Ref.	2.1	–
Models 2 and 3	–	Ref.	95.6
AUC	0.836	0.885	0.885
Difference in AUCs			
Models 1 and 2	Ref.	0.049	–
Models 2 and 3	–	Ref.	0
Regional level			
ICC (%)	67.7 (60.8–74.0)	68.0 (61.0–74.2)	20.5 (15.3–27.0)
MOR	12.3 (7.6–16.9)	12.4 (7.67–17.19)	2.41 (2.03–2.79)
PCV (%)			
Models 1 and 2	Ref.	− 1.1	–
Models 2 and 3	–	Ref.	87.8
AUC	0.707	0.810	0.810
Difference in AUCs			
Models 1 and 2	Ref.	0.103	–
Models 2 and 3	–	Ref.	0

Among 83,346 eligible patients, 30,343 (45.4%) were treated in the ICUs on the day of invasive mechanical ventilation initiation. Model 1: multilevel logistic regression with random intercepts for clusters. Model 2: multilevel logistic regression with patient-level covariates and random intercepts for clusters. Model 3: multilevel logistic regression with patient-level variables, cluster-level variables, and random intercepts for clusters

ICC, intraclass correlation coefficient; ICU, intensive care unit; MOR, median odds ratio; PCV, proportional change in variance; AUC, area under the receiver operating characteristic curve

In addition, Sakamoto et al. correctly pointed out that the “J045 Artificial ventilation” code includes manual ventilation (e.g., bag-valve-mask ventilation). However, manual ventilation that does not require subsequent invasive mechanical ventilation is rarely performed outside of CPR scenarios. Furthermore, the Japanese procedure code “J045 Artificial ventilation” has different medical fee points depending on the duration of artificial ventilation (Table 1). Therefore, patients for whom “J045 Artificial ventilation” was reimbursed for a short duration (e.g., 30 min) are likely to have received manual ventilation. This potential association warrants further investigation in future studies.

We greatly appreciate the opportunity to address the concerns raised by Sakamoto et al. regarding the method to identify patients requiring invasive mechanical ventilation. We plan to conduct a validation study to determine the reimbursement codes for invasive mechanical ventilation using the Japanese administrative claims data. Our ongoing research aims to improve the accurate understanding of the complex coding system and promote the appropriate utilization of Japanese administrative claims data.

## Data Availability

Not applicable.
